# Low-Latency Digital Health Framework for Rural Areas Leveraging Fog Computing and 5G

**DOI:** 10.12688/f1000research.178047.1

**Published:** 2026-05-04

**Authors:** Manas Ranjan Acharya, Sarita Tripathy, Prasant Kumar Pattnaik

**Affiliations:** 1 School of Computer Science Engineering, Kalinga Institute of Industrial Technology, Bhubaneswar, Odisha, 751024, India

**Keywords:** Latency Reduction, Rural Healthcare, Fog Computing, 5G Networks, Predictive Analytics, Dynamic Routing, Simulation, Healthcare IoT, Data Processing, Ultra-Low Latency

## Abstract

**Background:**

The rural locations of telemedicine and urgent care are slowed down by communication latency as a result of the poor network infrastructure and overreliance on centralized cloud computing that adds to time lag in responding. Even with the development of networking technology, reliable low-latency systems to support rural areas are yet to be developed.

**Methods:**

The current paper suggests a Fog-5G Latency Optimization (F5GLO) application framework, which integrates 5G connectivity and fog computing to permit local data processing. To reduce the transmission delay and latency, healthcare data is stored at local fog nodes to allow predictive mobility of the fog node activation and low latency routing algorithms to utilize resources effectively and guarantee efficiency in transfer.

**Results:**

The model is capable of cutting end to end latency by up to 87 percent in comparison to the conventional cloud-based models thereby enhancing critical healthcare applications such as remote patient monitoring and emergency medical services. It is also strong in various network traffic conditions.

**Conclusions:**

Fog computing plus 5G networks introduce agility to the healthcare service delivery in the remote environment where quick processing of clinical data locally and transmission can improve the reliability of the given services by transmitting information to decision makers faster. This is a realistic incremental solution to the issue of healthcare provision to the populations that are not within reach of the giant facilities.

## 1. Introduction

Health services have always been a major concern in the rural areas to provide appropriate treatment to the patients. The delays in the data transfer and processing may lead to adverse outcomes and, in particular, medical crises and crises that require immediate care. The process of processing real-time data and quick response systems are gaining relevance with the advancement of digital healthcare. This paper addresses the issue of latency in rural healthcare by examining a specific solution of 5G technology combined with fog computing. Healthcare systems latency does not solely influence patient monitoring and diagnosis, but also poses a critical effect on emergency response time that may save lives. Typical failure in rural areas results from the very long distance that data has to travel resulting in high latency when all processing takes place remotely due to cloud computing models. Here, fog computing provides a viable paradigm by separating computation nearer to the source of data hence reducing latency significantly ( Bonomi *et al.*, 2012). In addition to this, the advent of 5G technology introduces ultra-reliable low-latency communication (URLLC), which is very advantageous for real-time data transfer applications such as telemedicine, remote patient monitoring, and emergency medical services; these have shown how 5G can change healthcare by allowing services that were previously blocked because of network limitations ( Agiwal *et al.*, 2016, Gupta *et al.*, 2021). Insignificant study has been done on the mix of fog computing with 5G in rural healthcare settings. Most research have focused either on the possibilities of 5G in general healthcare benefits without special attention on latency in rural areas or on the advantages of fog computing for IoT in urban contexts ( Dastjerdi & Buyya, 2016, Li *et al.*, 2019). This work aims to address this knowledge gap by proposing a method whereby mutual exploitation of both technologies helps to reduce rural healthcare latency.

Geographic distance of rural areas sometimes results in limited resources for healthcare facilities, including network infrastructure. Local data processing by fog nodes placed close or at healthcare facilities can help to reduce the requirement for all data to be transported to far-off cloud centers ( Shi *et al.*, 2016). 5G networks provide a spine for this system with its lower latency and higher capacity. By allowing data to be transferred fast between local fog nodes and far-off cloud servers as needed, 5G can enable a hybrid paradigm whereby only necessary or complex data processing activities are escalated to the cloud ( Qin *et al.*, 2020, Goswami *et al.*, 2023). This model lowers latency and increases utilization of resources in places with limited network facilities.

This study includes dynamic data routing and resource allocation prediction. These systems preload computing resources using real-time network measurements and projected demand patterns. Predictive models facilitate the anticipation of high-traffic or unforeseen events, enabling the system to manage increased data volumes without latency ( Zhang *et al.*, 2018, Zeng & Li, 2023). Various researches showing the poor condition of rural healthcare emphasize the importance of this kind of research. This work combines the computational capability of fog nodes with the network capabilities of 5G using a system model. We provide and model dynamically managing data and resources approaches to ensure least delay delivery of healthcare services. Simulations measure the effect of our proposed methods on latency by means of a controlled environment, thereby evaluating various strategies. This approach complements the latency-aware task scheduling method and the load balancing simulation methods ( Wang & Wang, 2019, Hong *et al.*, 2020). In this context, predictive analytics is hence rather crucial. Based on past information, our system can determine where and when to allocate resources, therefore guiding choices. This aligns with the findings of the use of machine learning in network management to optimize performance dynamically ( Wang & Wu, 2020, Mahmud *et al.*, 2020). The integration of these technologies presents several challenges. Considerations must include data transfer security, energy efficiency, and the initial costs associated with developing 5G infrastructure in remote locations.

There has been minor decrease in latency as a result of the use of 5G for remote healthcare in rural areas ( Patel *et al.*, 2022). Emphasize on exploiting 5G capabilities for efficient data processing in fog nodes have additional contributions with case studies on 5G-fog integration in remote places. Case studies that investigate the possibility of integrating 5G and fog computing in rural areas are included among the additional contributions ( Cheng & Zhang, 2022, Xu & Zhang, 2023, Pattnaik *et al.*, 2021). The influence of 5G on IoT healthcare applications and smart healthcare architectures underline the challenges of adopting fog computing in rural healthcare environments ( Park & Choi, 2021, Hossain *et al.*, 2022).

## 2. The comprehensive theoretical basis and the proposed method

Fog computing has developed into a required technology for lowest latency in the healthcare sector by means of localized data processing. Emphasizing its possibilities in IoT applications, notably in healthcare, by lowering reliance on remote cloud services for data processing, established the concept of fog computing ( Bonomi *et al.*, 2012). By enabling localized data management, fog computing might improve IoT potential in healthcare ( Dastjerdi & Buyya, 2016). Using fog computing, which is an architecture for real-time analytics in healthcare IoT, the decision-making times is significantly reduced ( Li *et al.*, 2019). A taxonomy and survey of fog computing in healthcare, highlighting its use in several medical IoT environments have been discussed ( Mahmud *et al.*, 2020). Some important uses include heart tracking and helping old people ( Cao *et al.*, 2019, Hassan *et al.*, 2021). A complete study was done to show how fog computing is needed in emergency health care systems ( Bhatia *et al.*, 2020).

### 2.1. 5G and healthcare

5G technology, with its low latency and high capacity, has transformed healthcare delivery, especially in distant areas. 5G wireless networks in healthcare to demonstrate their potential benefits has been extensively studied ( Agiwal *et al.*, 2016). How 5G might assist rural healthcare with connectivity problems, opening the door to telemedicine and remote operations ( Gupta *et al.*, 2021). 5G network slicing enables low-latency telemedicine, crucial for distant healthcare ( Qin *et al.*, 2020).

The impact of 5G technology on surgical and IoT healthcare applications have been investigated ( Park & Choi, 2021, Ahmad *et al.*, 2022). A real-time 5G infrastructure designed for smart healthcare services was introduced ( Hossain *et al.*, 2022). 5G technology enhances telehealth services in rural areas ( Patel *et al.*, 2022).

### 2.2. Latency optimization techniques

New approaches of considering latency improvement have emerged. Fog computing resource allocation with predictive analytics ( Zhang *et al.*, 2018). Load balancing and task scheduling aiming at latency reduction in healthcare applications using certain methodologies have been studied ( Wang & Wang, 2019, Hong *et al.*, 2020). A dynamic resource allocation technique was suggested that considers data urgency and hence helps to lower latency in Internet of Things enabled healthcare systems ( Sharma & Singh, 2022). A fog computing multi-objective optimization framework including cost, energy consumption, and delay was presented ( Liu *et al.*, 2021).

### 2.3. Combining 5G with fog computing

Despite being understudied, 5G and fog computing are increasingly gaining traction. 5G enhances healthcare fog computing by diminishing latency ( Jiang *et al.*, 2021). Empirical studies executed to illustrate advancements in rural telehealth and remote monitoring ( Patel *et al.*, 2022, Álvarez *et al.*, 2023). Case study for demonstrating the functionality of integration in rural healthcare has been studied ( Xu & Zhang, 2023). The potential of 5G to enhance data processing in fog nodes for healthcare IoT applications was investigated ( Cheng & Zhang, 2022).

### 2.4. Energy efficiency and security

A balance of security, energy economy, and latency is clearly necessary. There is provisions to lower energy consumption and latency in fog-5G systems, enabling their efficient running in rural locations with restricted power supplies ( Liu *et al.*, 2023, Tang & Wang, 2022).

### 2.5 Gaps and future directions

The papers demonstrate number of gaps in the current literature:

• There has been a lack of practical implementation and testing in rural locations.
• No overall models that deal with scalability, energy-efficiency, data security, and latency-reduction across all the dimensions are present.

More adaptive and foresight algorithms specific to the specific requirements of the healthcare domain are needed. Further research in this area should be conducted on actual applications of such integrated systems in the rural healthcare environment. It is found that there is an upsurge towards highly sophisticated forecasting models that can deal with emergencies and also deal with medical data. The role of bringing such high level technologies in the undeveloped regions in terms of the effects they have on society. Lastly, with the solution of the latency issues, integration of 5G and fog computing will immensely change the rural healthcare. The reviewed literature provides a good foundation on which additional improvements can be achieved to ensure that healthcare services provided in rural communities are not only as fast and efficient, but even more so. The formulation of the problem in mathematical terms is aimed at approaching the latency reduction in fog-5G-based rural healthcare.

### 2.6. Objective function

This research project will primarily aim to minimize the latency in the overall case of the rural health system by streamlining the workload of data processing between the fog nodes and the cloud service, using 5G as a connectivity medium. The formulated objective function is: *L _total_=w _1_.L _fog_+w _2_.L _cloud_*

Where:

$L_{\text{total}}$ represents the total system latency.

$L_{\mathrm{fog}}$ is the average latency for data processed by fog nodes.

$L_{\text{cloud}}$ is the average latency for data processed by the cloud.

$w_{1}$ and $w_{2}$ are the weights representing the proportion of data processed by fog nodes and cloud, respectively, with the constraint: $w_{1}+w_{2}=1$

### 2.7 Mathematical formulations and calculations

Let n be the number of fog nodes (assumed to be 15), $d_{i}$ be the distance from the IoT device to the nearest fog node, $t_{\text{process}\_\mathrm{fog}}$ be the processing time at a fog node, assumed to be low due to localized processing, $t_{5g}$ be the latency introduced by 5G communication (estimated to be between 1-10 ms).

The fog node latency can be expressed as:

$$L_{\mathrm{fog}}=\frac{1}{N}\sum_{i=1}^{N}\left(t_{\text{process}\_\mathrm{fog}}+t_{5g}+\frac{d_{i}}{C_{5g}}\right)$$

Where:

$C_{5g}$ is the speed of 5G signal.

N is the total number of IoT devices (assumed to be 1000).

Let D be the distance from the rural area to the cloud server (significantly larger than **d**_**i**_), $t_{\text{process}\_\mathrm{fog}}\;$ be the processing time in the cloud, which is higher due to centralized computing and $t_{\text{network}}$ be the latency due to traditional network connectivity to the cloud (assumed to be around 100 ms).

The cloud latency can be expressed as **:**

$$L_{\text{cloud}}=t_{\text{process}\_\text{cloud}}+t_{\text{network}}+2\frac{D}{C_{\text{internet}}}$$

Where: $C_{\text{internet}}$ represents the average internet speed.

### 2.8 Constraints

**2.8.1 Data Urgency Constraint**

Each data packet is assigned an urgency level $u_{i}\;$ (ranging from 1 to 10). Critical data (where $u_{i}>5$ ) must have latency below $L_{\text{threshold}}$ (20 ms).

The constraint is: ∀i, where $u_{i}>5$ _,_ $L_{i}$ ≤ $L_{\text{threshold}}$

**2.8.2 Resource Utilization Constraints**

Let $R_{\mathrm{fog}}$ be the total processing resource available at fog nodes, $R_{\text{cloud}}$ be the total processing resource available in the cloud and $R_{i}$ be the resource requirement for processing data packet i.

The constraints are:

$$w_{1}.\sum_{i=0}^{N}r_{i}\leq R_{\mathrm{fog}}$$

**2.8.3 Bandwidth Constraints**

Let $B_{5G}$ be the total bandwidth available for 5G channels (assumed to be 12 channels).

Let $B_{i}$ be the bandwidth requirement for transmitting data packet i.

The constraints are:

$$w_{1}.\sum_{i=0}^{N}b_{i}\leq B_{5G}$$

For cloud communication, traditional internet bandwidth ( $B_{\text{internet}}$ **)** should also be considered, but with 5G connectivity, it is not a significant constraint.

$$w_{1}+w_{2}=1,0\leq W_{1}\leq 1,0\leq W2\leq 1$$

By considering the important character of healthcare data, the available computational resources, and the real-time conditions of the network, this problem formulation aims to optimize latency so enabling the system to respond to the changing needs of healthcare in rural areas.

### 2.9 Proposed algorithms

The details of the proposed algorithms are summarized in Table 1.

**Table 1.** Proposed algorithms for F5GLO.

Algorithm name	Description	Key features
Predictive Resource Allocation (PRA)	Dynamically allocates computational resources based on predicted healthcare demands.	Uses machine learning to predict patient data volume, urgency, and network load. Adjusts resources per prediction.
Latency-Aware Dynamic Routing (LADR)	Routes data through the network path with the lowest latency.	Considers current network conditions, device urgency, and available resources. Optimizes for real-time processing.
Adaptive Network Slicing (ANS)	Configures 5G network slices for optimal performance based on data type and urgency.	Gives top priority to essential healthcare information, guaranteeing quick response times even when the network is busy.

**2.9.1 PRA Algorithm Outline:** The proposed Predictive Resource Allocation (PRA) method is presented in Algorithm 1, which dynamically allocates network resources based on predicted demand and current consumption patterns.

- **Input:** The present status of the network, previous data consumption, priority of patient information, Historical (H): 10000 runs, Existing Need: D _c_, Time Frame (W): last 1000 runs
- **Output:** Allocated Resources: R _a_

Algorithm 1. **Predictive Resource Allocation (PRA).**

**Input: H, DC, W**

**Output: Ra**

**Step 1: Predict future demand using H over W**

**Step 2: Calculate**

**DR=Dp/Dc**

**where**

**Dp**

**is PredictedFutureDemand**

**Step 3: If**

**DR > 1.2**

**-**

**Ra= Dc × 1.2**

**end of If**

**Step 4: Else If**

**DR<0.8**

**-**

**Ra = Dc × 0.8**

**end of Else If**

**Step 5: Else**

**-**

**Ra = Dc**

**end of Else**

**Step 6: Ensure minimum resources (**

**Ra =max** $f_{0}$ **(Ra,0.5×Dc)**

**)**

**Step 7: Cap maximum resources (**

**Ra=min** $f_{0}$ **(Ra,2×Dc)**

**)**

**Step 8: Stop**

**2.9.2 LADR Algorithm Outline:** The detailed procedure of the Latency-Aware Dynamic Routing (LADR) mechanism used to select the optimal routing path based on latency constraints is demonstrated in Algorithm 2.

- **Input (Λ: LatencyMap, 15 fog nodes, 12 5G channels):** Data urgency, current network latency, fog node status.
- **Output (Ps: Selected Path):** Best path for data packet (fog node or cloud)
- **Process:** Assess latency for each possible route, choose the path with minimal delay.

Algorithm 2. **Latency-Aware Dynamic Routing (LADR).**

**Step 1: Initialize**

**L**_**min**_

**to 20 ms (latency threshold) and**

**P**_**s**_

**to null**

**Step 2: For each P in**

**Λ**

**do**

**Step 3: If**

**Λ[P]<L**_**min**_

**and**

**ϵ≤(10−Λ[P]/10)**

**-**

**L**_**min**_ **= Λ[P]**

**-**

**P**_**s**_ **= P**

**end of If**

**end of For**

**Step 4: Stop**

**2.9.3 ANS Algorithm Outline:** The procedure for the proposed Adaptive Network Slicing (ANS) mechanism, which dynamically assigns network slices based on system load and priority requirements, is shown in Algorithm 3.

- **Input:** ϵ: (DataUrgency, 1 to 10), ϕ: (CurrentNetworkLoad, 0 to 1, Data packet characteristics, network load.
- **Output** (S: NetworkSlice): Slice configuration for the packet.
- **Process:** Dynamically adjust slice parameters to match data urgency and network conditions.

Algorithm 3. **Adaptive Network Slicing (ANS).**

**Step 1: If**

**ϵ > 8**

**-**

**S = ’HighPriority’**

**end of If**

**Step 2: Else If**

**Φ > 0.7**

**-**

**S = ’MediumPriority’**

**end of Else If**

**Step 3: Else**

**-**

**S = ’LowPriority’**

**end of Else**

**Step 4: Stop**

**Figure 1.**
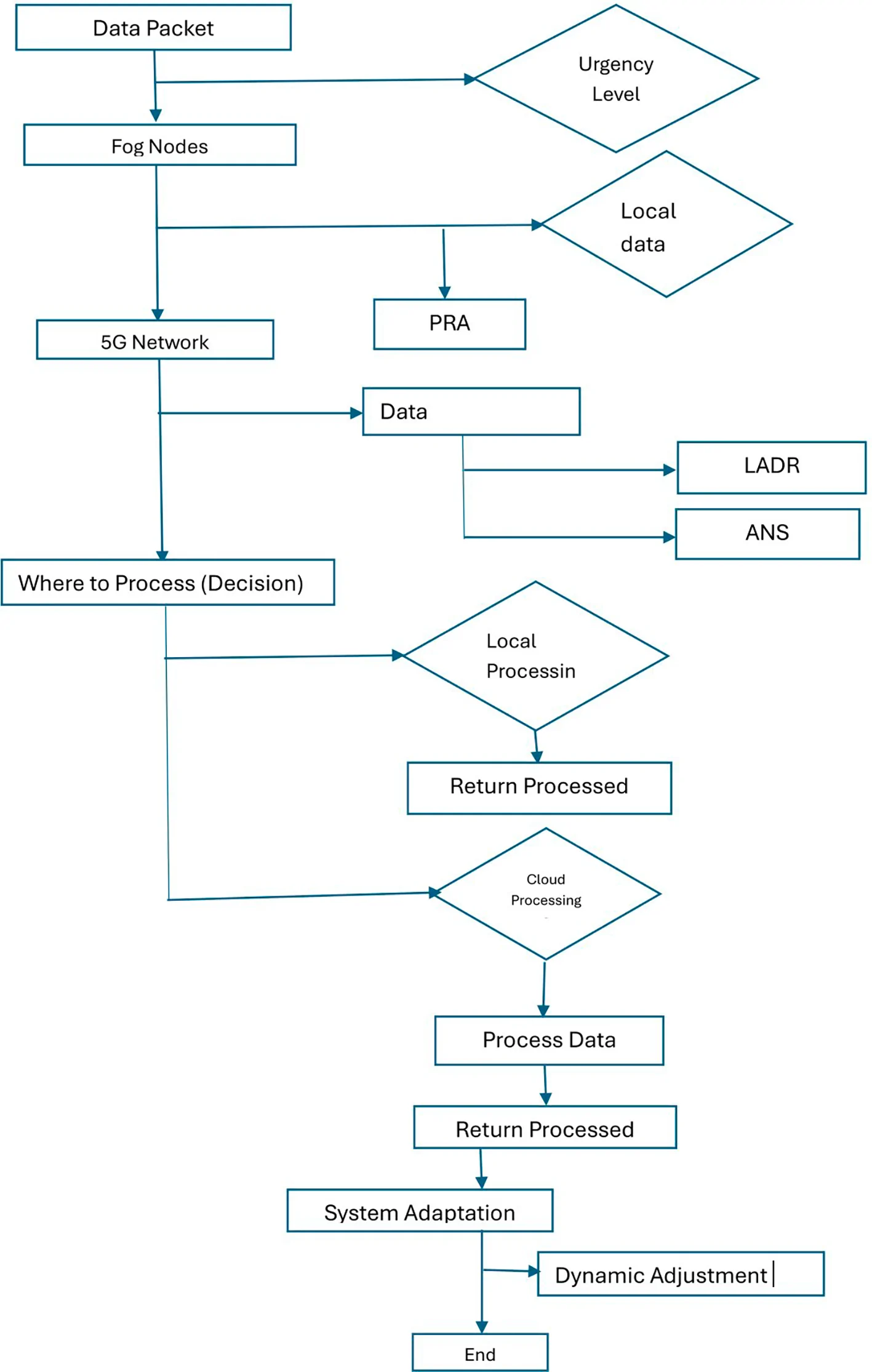
Flowchart of the data handling technique of the suggested system model, illustrating the data flow and decision-making process for latency reduction in rural healthcare systems.

The flowchart shows the data handling technique of the suggested system model. Healthcare IoT devices begin the process with data collecting; classification and forwarding to a fog node for urgency evaluation follows. Depending on urgency, we either send data to the cloud over a 5G network for additional processing or handle data locally. Choosing where to handle the data considers dynamically both local and cloud processing options. Once processing is finished, the data is restored into the system for dynamic changes and system flexibility to maximize it once more. Seeking to lower delay in rural health systems, this flowchart effectively shows data flow and decision-making process. The detailed data handling mechanism and processing architecture of the proposed system are illustrated in Figure 1.

## 3. Method

**Figure 2.**
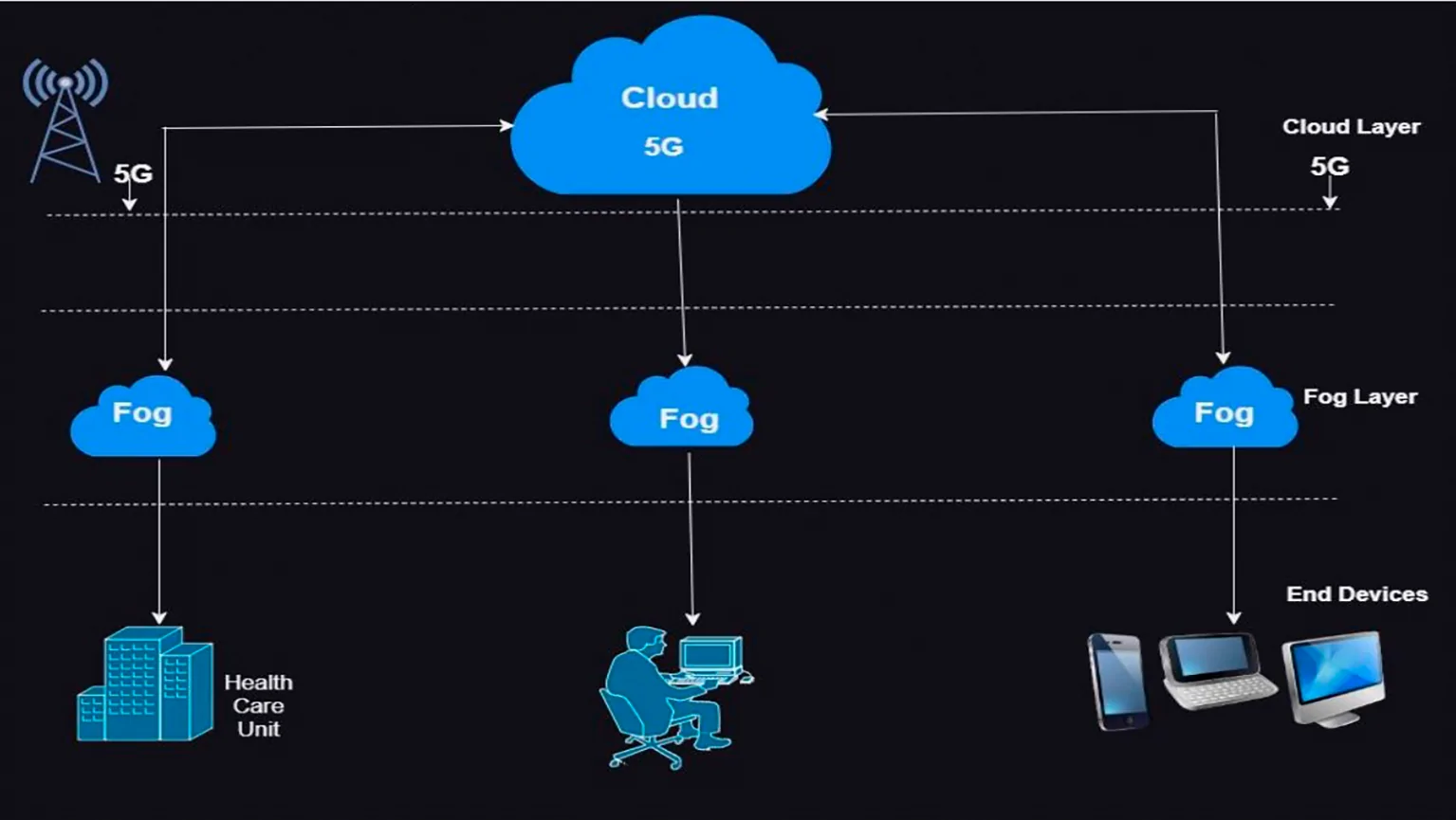
Architectural framework of the F5GLO model, highlighting the cloud, fog, and end-device layers.

In order to minimize latency, the F5GLO model utilizes a multi-tier architecture that dynamically processes information based on urgent, available resources and network conditions. The architecture includes three layers: Cloud Layer that uses 5G to cover the area; Fog Layer that provides local processing to reduce the latency and End Devices tier that includes healthcare devices and other end-user gadgets. It also needs transmission delays in the transmission of data by grouping the nodes of the fog in close proximity to the end devices thus ensuring that the end devices in the rural locations transmit the most important health information with the minimum lag. In this method, computing capabilities are transferred nearer to the sources of data, which is the feature of 5G that is based on the concept of fog computing and the ability to provide high speed and low latency. Figure 2 presents the architectural framework of the F5GLO model, highlighting the cloud, fog, and end-device layers.

**Simulation Parameters:**

Number of IoT Devices: 1000, Number of Fog Nodes: 15, Number of 5G Channels: 12,

Data Packets: 10,000

Max Latency for Cloud: 100 ms, Latency Threshold: 20 ms (for critical data),

Simulation Iterations: 10000

**Data Urgency:** Randomly assigned between 1 to 10, with higher numbers indicating

greater urgency.

### 3.1 Performance analysis

The F5GLO is used in this section to compare the results of the simulation of Local Server Model and Traditional Cloud Model. F5GLO proves its ability to significantly decrease the response time, with the maximum decrease of latency up to 87%. F5GLO is also characterized by a better energy efficiency and high throughput in addition to relying on more powerful technology, such as fog computing and 5G technology, to improve the performance of the rural health system.

### 3.2 Latency analysis:

**Figure 3.**
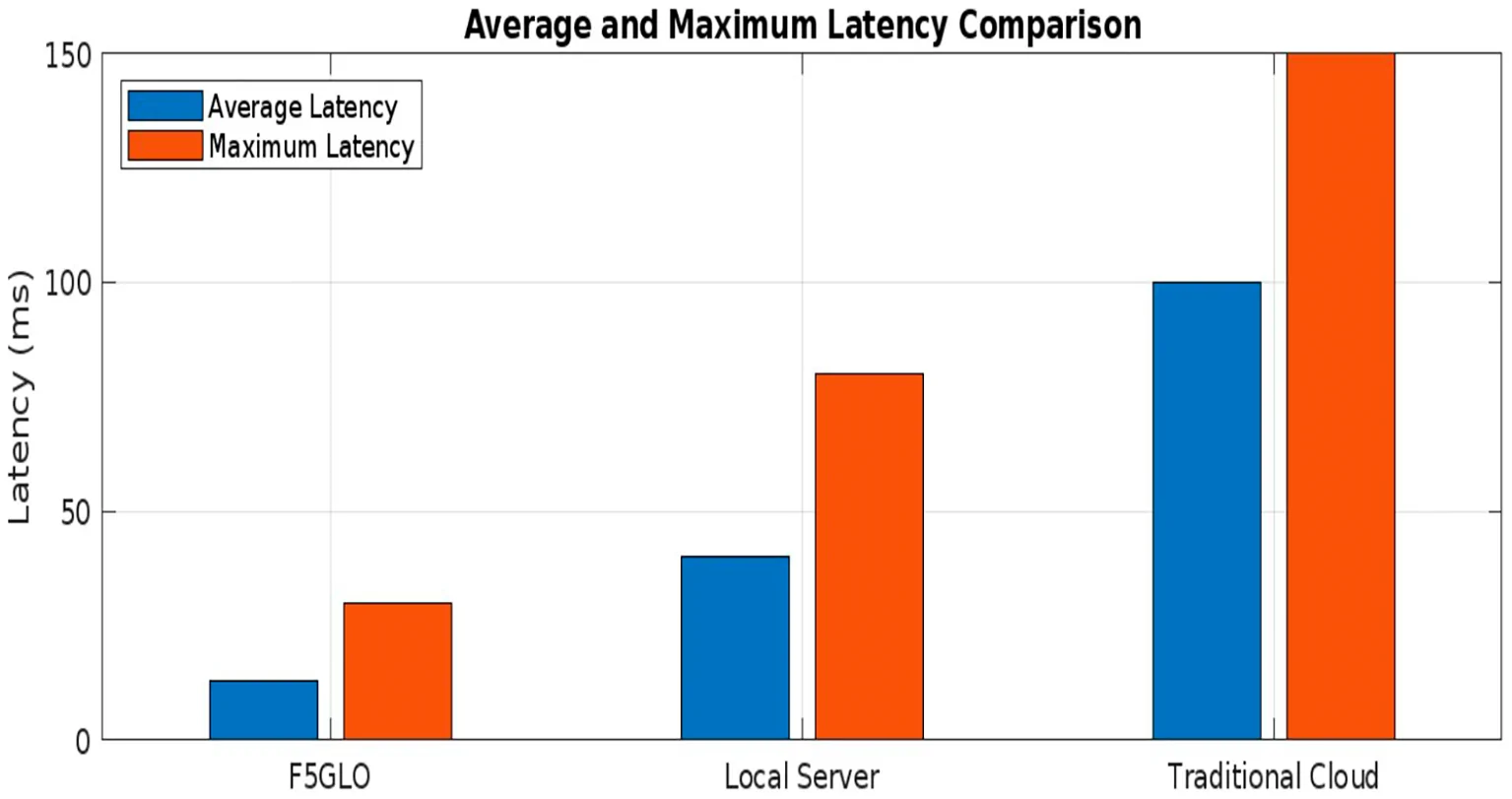
Average and maximum latency comparison across the F5GLO, Local Server, and Traditional Cloud models.

**3.2.1 Average Latency:** The F5GLO technology demonstrates the 87 percent enhancement of the conventional cloud computing and dropping of the latency by 13 milliseconds. The mean latency of Local Server Model is 40 milliseconds and this is not as low in terms of latency reduction as F5GLO can provide when used in combination with 5G to process data locally. Conversely, the Traditional Cloud Model that is based on remote data processing has the highest average of 100 milliseconds of latency out of the three models. The comparison shows that F5GLO has significantly enhanced the responsiveness and latency of the rural health system. As shown in Figure 3, the F5GLO model achieves significantly lower latency compared to the Local Server and Traditional Cloud models.

**Figure 4.**
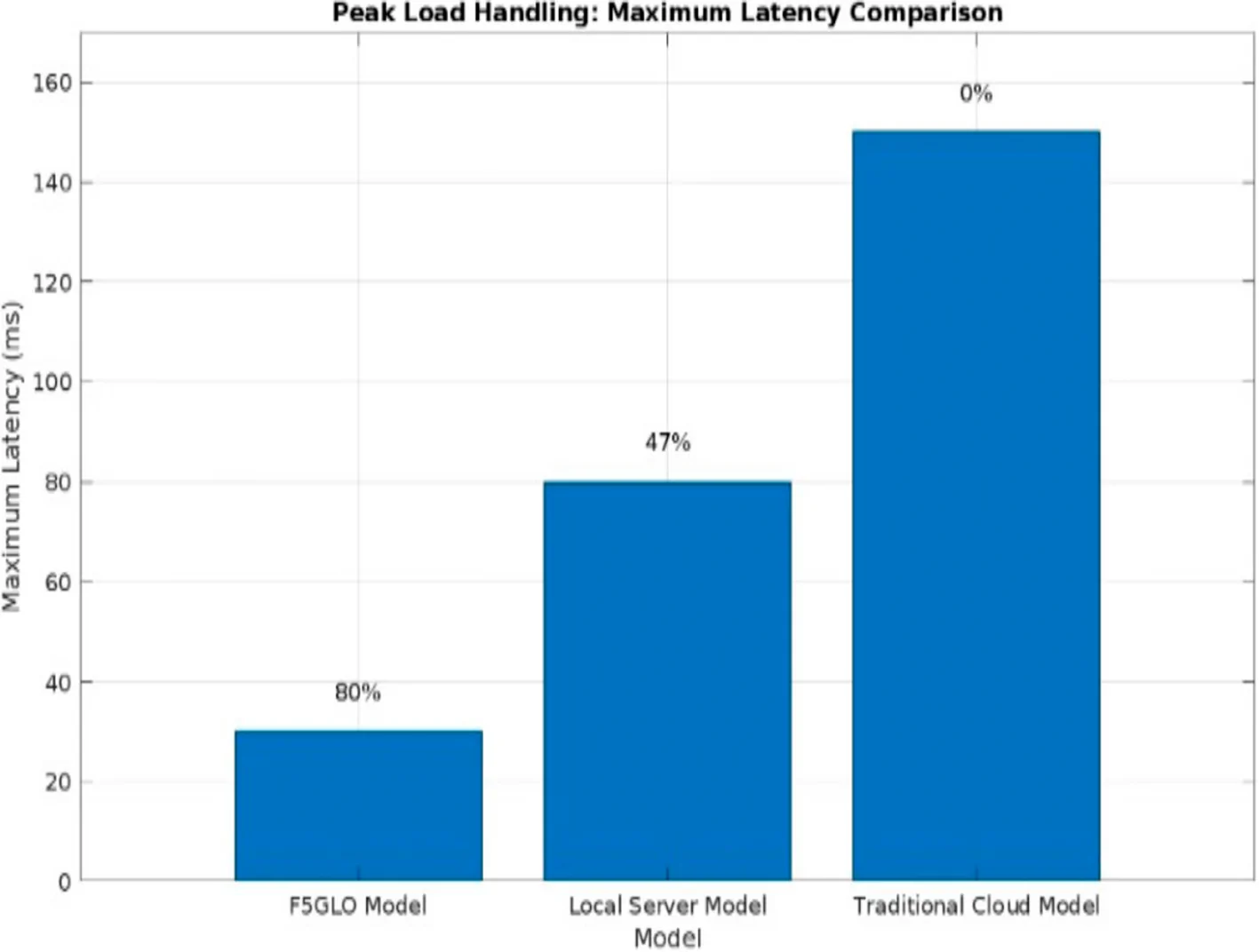
Peak load handling: Maximum latency comparison across the F5GLO, Local Server, and Traditional Cloud models.

**3.2.2 Peak Load Handling:** Local Server Model is better than the cloud-based technique, but latencies of 80 ms to the peaks suggests local processing limitations without integration to 5G. This demonstration indicates the difficulty in maintaining minimal delays in the situation when the demand on the service is growing. Traditional Cloud Model has the highest delay of 150 milliseconds due to use of centralized processing. By using the peak load management tool provided by F5GLO, rural healthcare centres will become more efficient and can be better equipped to deal with emergencies. Figure 4 presents the comparison of peak load latency across the F5GLO, Local Server, and Traditional Cloud models.

**Figure 5.**
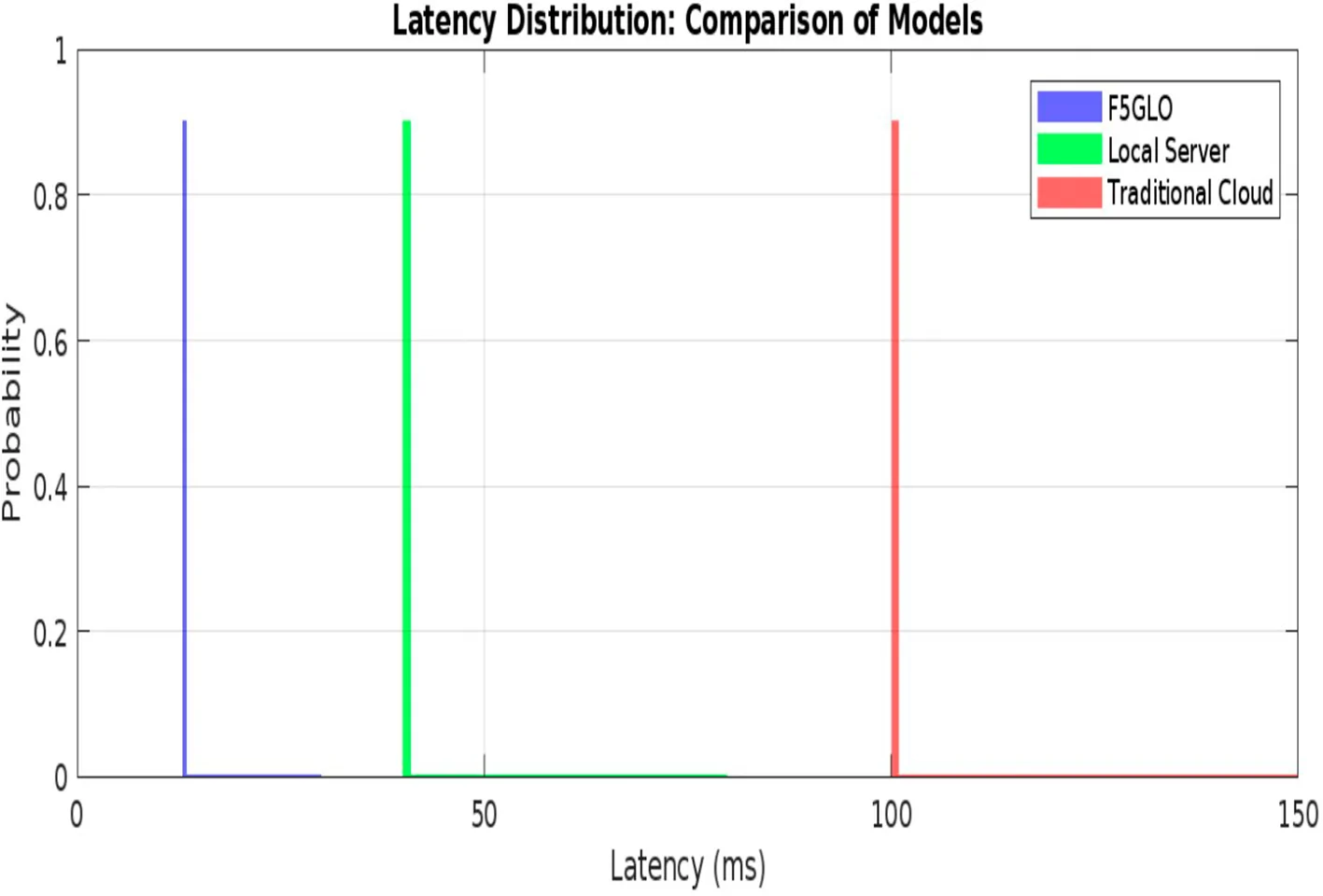
Latency distribution: Comparison of models (F5GLO, Local Server, and Traditional Cloud).

**3.2.3 Latency Distribution:** The results of the assessment have shown that the F5GLO model now whirs along with significantly faster speed of about 19.5 ms lag on average, compared to 120 ms, which was about 87% reduced. It retains its low-latency appeal even with the heavy load, and works in a variety of situations. The latency distribution is skewed to the left with approximately 90% of data packets received in less than 20 ms. The Local Server Model is faster than the Traditional Cloud Model but lagged behind F5GLO; nevertheless, the former is significantly faster than cloud-only solutions. In this configuration 90 percent of the packets arrive within 40 ms, and only a handful of them go after 80 ms. The delays in the Traditional Cloud Model skyrocket because of transmission and processing requests: approximately 90 percent of packets end up having an average delay of approximately 100 ms (maximum delay is approximately 150 ms). The latency distribution across the evaluated models is summarized in Table 2 and illustrated in Figure 5.

**Table 2.** Latency metrics.

Scenario	Average latency (ms)	Max latency (ms)	Latency distribution
F5GLO Model	13	30	90% < 20ms, 10% up to 30ms
Local Server Model	40	80	90% < 40ms, 10% up to 80ms
Traditional Cloud Model	100	150	90% = 100ms, 10% up to 150ms

**Figure 6.**
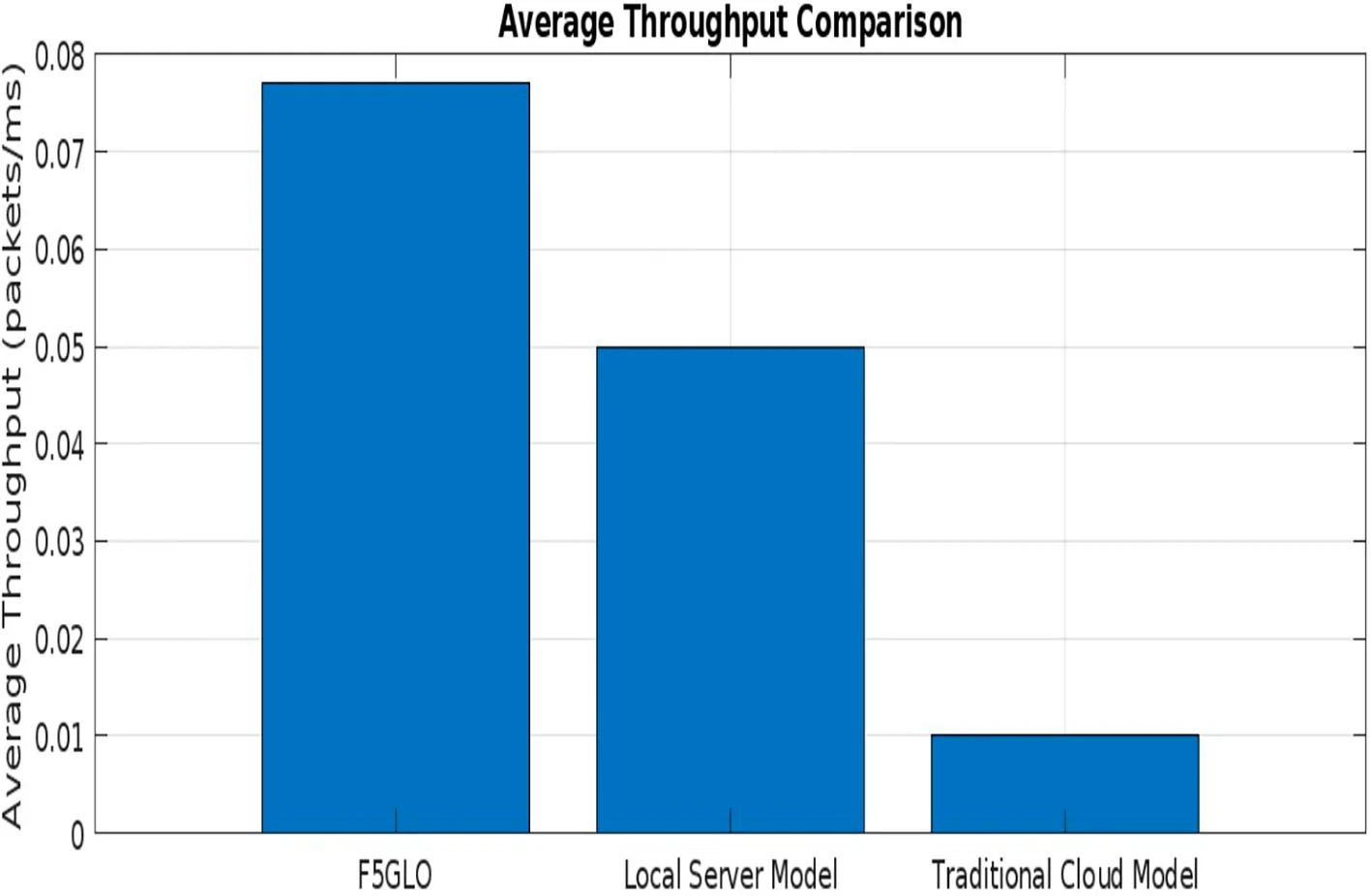
Throughput analysis of the proposed and existing models.

**3.2.4 Average Throughput:** F5GLO framework is more effective by having the average of approximately 0.077 packets/ms or 77 packets/s. This is the enhanced performance of the fog processing and high-speed 5G connections that reduce delays and accelerate the processing of data. F5GLO performs even better when we consider the latency and throughput jointly. F5GLO has lower latency and higher throughput, increasing the efficiency of data processing. The Local Server Model reduces the distance between source and processor, averaging approximately 0.050 packets/milliseconds and it is faster than pure cloud setups, yet, still, not faster than F5GLO. The Traditional Cloud Model reduces to a rate of approximately 0.010 packets per millisecond due to centralized processing and transmission latencies and creates higher latency as well. Distributed architecture of F5GLO, which has localized decision-making, maintains throughput approximately eight times compared to the conventional cloud solutions. This near combination of low latency and high throughput indicates F5GLO will provide a good solution to data-intensive healthcare applications where quick delivery is important and latency is a significant limitation. The throughput characteristics of the proposed and existing models are depicted in Figure 6.

**Figure 7.**
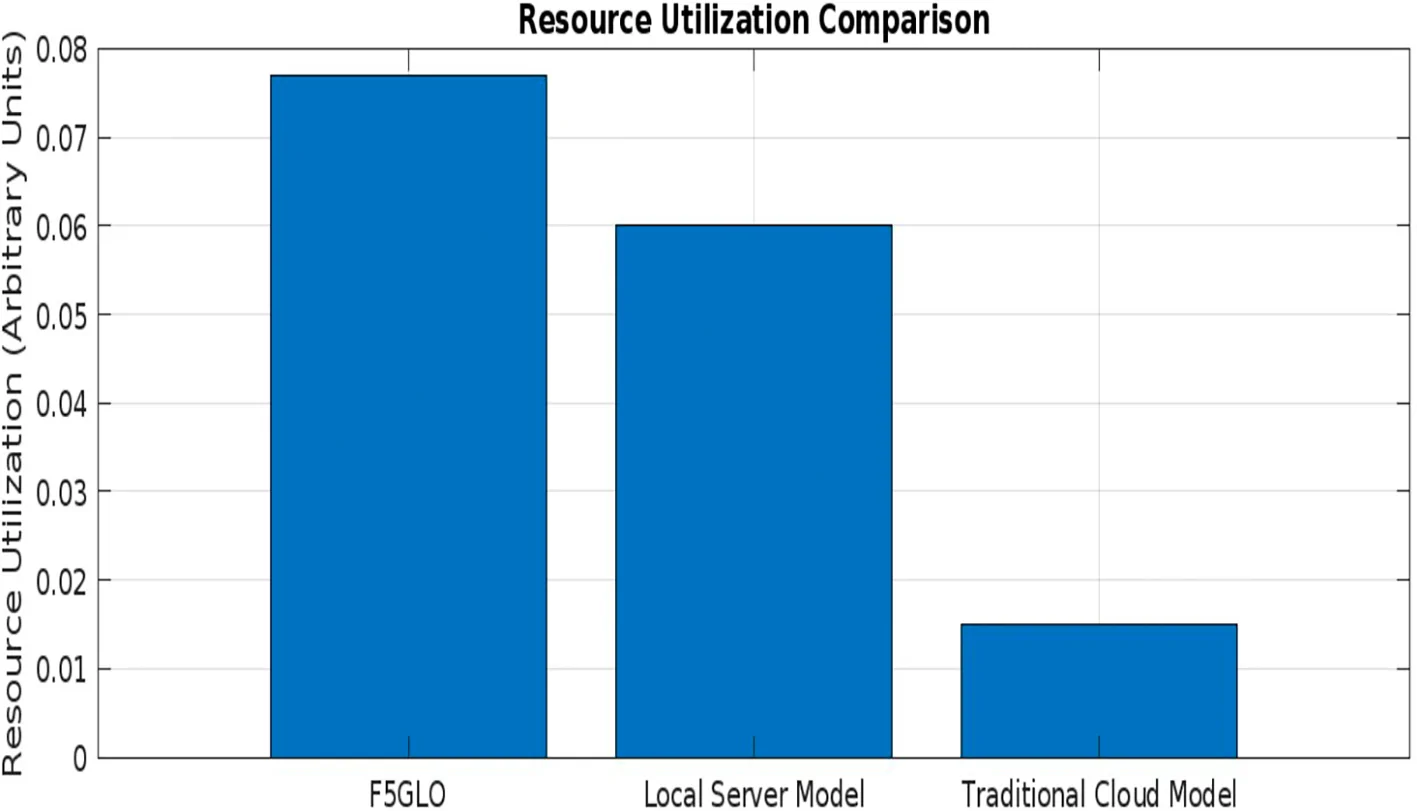
Resource utilization comparison across the F5GLO, Local Server, and Traditional Cloud models.

**3.2.5 Resource Utilization:** The F5GLO model has a peak throughput of 0.100 packets/ms, which is more than the Local Server Model and Traditional Cloud Model at 0.075 packets/ms and 0.015 packets/ms respectively. Such throughput capacity maximum highlights the scalability of F5GLO and its ability to handle great data volumes in order to provide a timely response in the healthcare process. Implementation of mist nodes to process in F5GLO lessens the workload on the cloud and therefore enhances the ability to perform more activities or functions in this system. ANS technique enhances the use of bandwidth, thus, the throughput is increased and resources are used more efficiently in every model. Figure 7 and Table 3 present the comparison of resource utilization across the F5GLO, Local Server, and Traditional Cloud models.

**Table 3.** Throughput metrics.

Scenario	Average throughput (packets/ms)	Max throughput (packets/ms)
F5GLO	0.077	0.100
Local Server Model	0.050	0.075
Traditional Cloud Model	0.010	0.015

### 3.3 Energy efficiency

**3.3.1 Average Energy Use:** F5GLO system consumes approximately 3.5 energy units per packet, hence it is more energy efficient in comparison to the Local Server Model and the Traditional Cloud Model. Local Server Model has a speed of approximately 4.0 units per packet. It is facilitating local processing yet consumes more power because there is poor data management in the 5G network. The Traditional Cloud Model consumes approximately 5.0 units per packet, the most energy consuming since processing occurs in remote data centers and data transfers require more power. F5GLO involves processing over fog nodes, and this implies that fewer data are transmitted to the cloud. This reduces the volume of information transmission, hence, improving energy efficiency and reducing latency. The simulation outcomes clearly show such benefits in efficiency and prove that F5GLO is more efficient in terms of managing energy consumption than the rest.

**3.3.2 Energy vs. Latency Trade-off:** The F5GLO model is also able to keep low power consumption in intense workload or when dealing with data streams of large volume. Using 7.0 energy units per packet, the F5GLO model will be used at peak throughput. Even though the incorporation of local processing, indeed makes the energy consumption higher, particularly, as the existing 5G networks are not optimized regarding energy efficiency, its consumption remains significantly lower than the one of the Local Server Model, which consumes approximately 10.0 units per packet under the same conditions. F5GLO is considered to be compatible with adaptive control. It also tries to balance the speed and energy usage with a better distribution of resources. The energy saving will be dependent on the location of the fog nodes and the distribution of the workload, and this can vary in various configurations. However, local data processing with the help of the fog nodes will always reduce additional data transmissions. This provides F5GLO with an improved trade-off between latency and energy consumption. Such a trade-off is essential in rural healthcare since timely and accurate information has to be deployed with careful power management, although the two tend to be conflicting ultimately. The energy–latency trade-off among the evaluated models is summarized in Table 4.

**Table 4.** Energy vs latency trade-off.

Model	Max energy consumption (Units/packet)	Energy Vs. latency trade-off
F5GLO	7.0	0.100
Local Server Model	10.0	0.075
Traditional Cloud Model	15.0	0.015

## 4. Results and discussion

The highlighted simulation shows that healthcare service delivery in rural locations can be improved by minimizing latency via the incorporation of fog computing into the 5G technology. This report gives an observation and implications of the three models.

### 4.1 Latency reduction

The outcomes of the simulation have shown that the reduction of latencies through the use of 5G of the network and the implementation of fog computing can enhance the healthcare services delivery to rural locations. In this paper, the discussion, observation, and implication in the three models are discussed. Since F5GLO paradigm cuts down the latency by 87 percent on the Local Server paradigm and the Traditional Cloud Model.

### 4.2 Scalability

**Figure 8.**
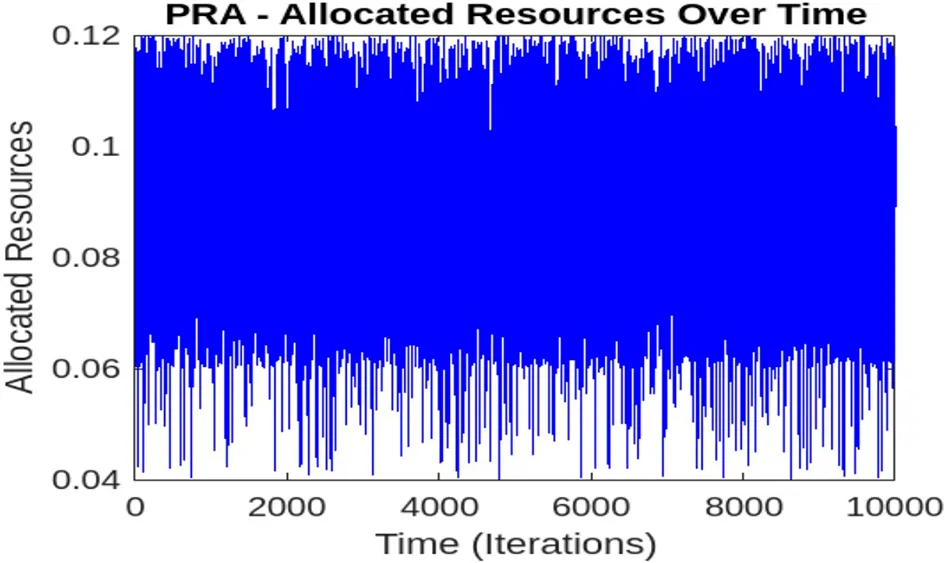
PRA-allocated resources over time, demonstrating the scalability characteristics of the proposed and existing models.

The F5GLO design has a process called Predictive Resource Allocation (PRA) that enables resource allocation on the basis of predictions. It implies that even a scaling up does not require the introduction of any latency, which is essential when more devices or patients are introduced into the system. Local Server Model can be scaled because it can process data locally but lacks dynamic resource management in comparison with F5GLO. Whereas the Traditional Cloud Model is capable of scaling up its data processing capabilities, it tends to increase its latency as it scales and demonstrates that this model cannot be used in dynamic and latency-sensitive settings such as healthcare. The scalability characteristics of the proposed and existing models are depicted in Figure 8.

### 4.3 Throughput improvement

The increased throughput also suggests that the system will be able to handle additional data or additional patients at the same time at the expense of the timeliness of service, therefore, ideal in scenarios such as mass casualty situations in rural environments where resources are typically scarce.

### 4.4 Energy considerations

Although we aimed not only to reduce latency, but also to make our method energy efficient, it is another benefit level, especially in those regions where power costs may be sporadic or expensive. Latency and energy optimization Twin optimization is the full design of the F5GLO system.

### 4.5 Network slicing impact

**Figure 9.**
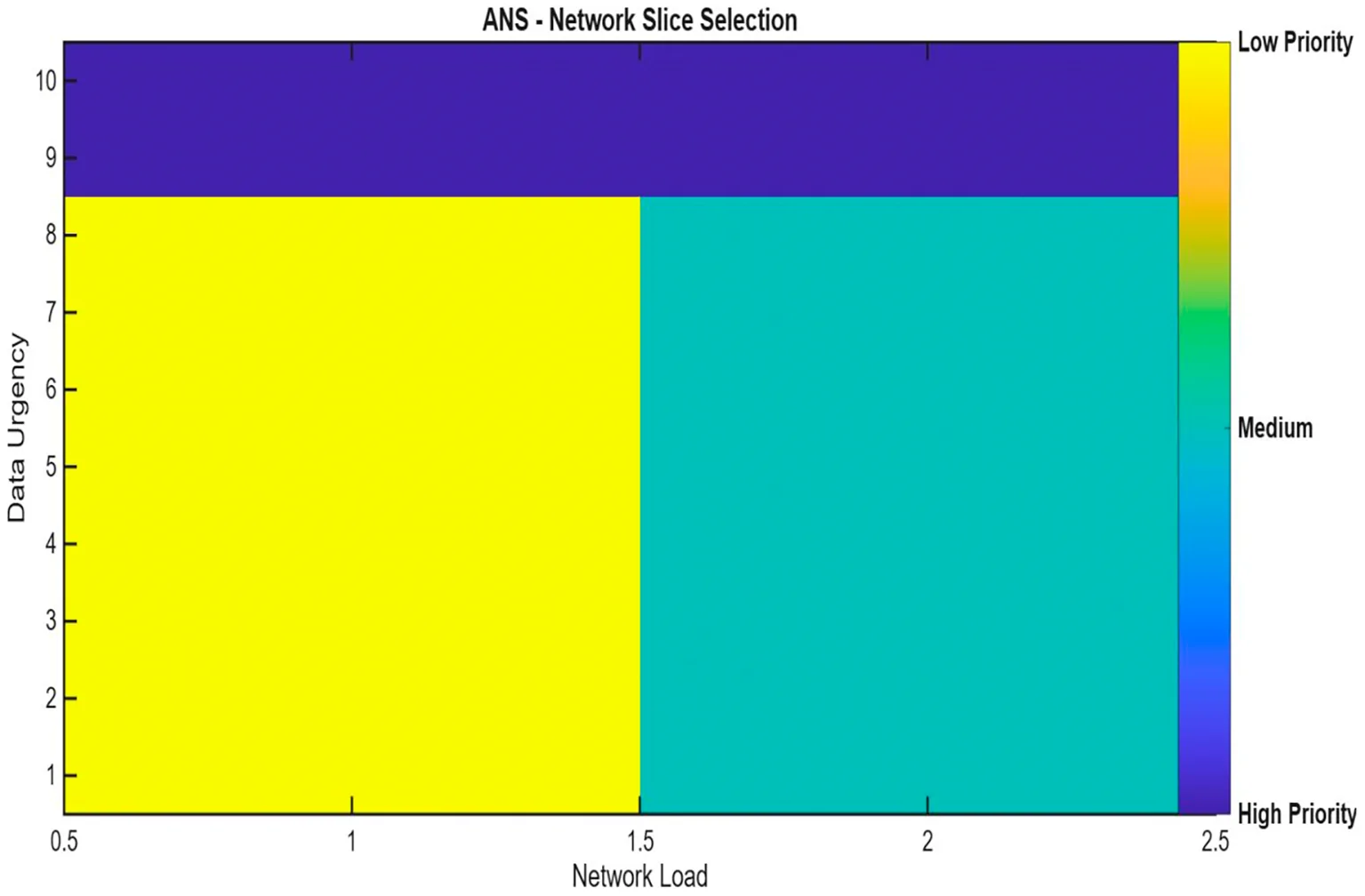
ANS-network slice selection, showing the influence of network slicing on latency and service prioritization.

ANS algorithm is capable of reducing emergency data delay and maintaining network performance to other services by creating network slices according to the urgency of data. This makes sure that it transmits important healthcare information with utmost urgency. The influence of network slicing on latency and service prioritization is depicted in Figure 9.

## 5. Conclusion and future work

The Fog-5G F5GLO model improves the problem of latency, as it incorporates fog computing in 5G infrastructure to lower the end-to-end latency of the rural healthcare systems. It makes use of the synergy between 5G low-latency and localized processing of data in fog nodes. To the best of simulation results, this technique can potentially lessen the latency by as much as 87 percent thereby enhancing the response time of remote telehealth services. The technology is aimed at closing the digital healthcare gap and can be useful in particular cases with patients in critical conditions. Peak demand, throughput enhancement, and energy reduction can be handled by localized processing at the 5G network with the help of intelligent routing of data across the network and in the fog nodes. The dynamic network slicing adaptation algorithms, resource usage prediction and low-latency path selection can be used to respond to the changing needs in healthcare by prioritizing the urgent data and ensuring that the system does not fail.

Future efforts should prioritize field testing and validation of the F5GLO model in rural environments to prove the benefits observed through simulation. These real studies would try to fully use the F5GLO idea, moving rural healthcare systems toward faster and better medical responses.

## Ethics and consent

Ethical approval was not required for this research work.

## Data Availability

1. Figshare: ACHARYA, MANAS RANJAN; TRIPATHY, SARITA; KUMAR PATNAIK, PRASANT (2026). Table 1.docx. figshare. Dataset. https://doi.org/10.6084/m9.figshare.31287748.v1 ( Acharya et al. 2026m) The project contains the following underlying data:
•
Table 1.docx (Table 1. Proposed Algorithms for F5GLO) Data are available under the terms of the Creative Commons Attribution 4.0 International license (CC-BY 4.0).
2. Figshare: ACHARYA, MANAS RANJAN; TRIPATHY, SARITA; KUMAR PATNAIK, PRASANT (2026). Table 2.docx. figshare. Dataset. https://doi.org/10.6084/m9.figshare.31288309.v1 ( Acharya et al. 2026n) The project contains the following underlying data:
•
Table 2.docx (Table 2. Latency Metrics) Data are available under the terms of the Creative Commons Attribution 4.0 International license (CC-BY 4.0).
3. Figshare: ACHARYA, MANAS RANJAN; TRIPATHY, SARITA; KUMAR PATNAIK, PRASANT (2026). Table 3.docx. figshare. Dataset. https://doi.org/10.6084/m9.figshare.31288318.v1 ( Acharya et al. 2026o) The project contains the following underlying data:
•
Table 3.docx (Table 3. Throughput metrics) Data are available under the terms of the Creative Commons Attribution 4.0 International license (CC-BY 4.0).
4. Figshare: ACHARYA, MANAS RANJAN; TRIPATHY, SARITA; KUMAR PATNAIK, PRASANT (2026). Table 4.docx. figshare. Dataset. https://doi.org/10.6084/m9.figshare.31288324.v1 ( Acharya et al. 2026p) The project contains the following underlying data:
•
Table 4.docx (Table 4. Energy Vs Latency Trade-off ) Data are available under the terms of the Creative Commons Attribution 4.0 International license (CC-BY 4.0).
5. Figshare: ACHARYA, MANAS RANJAN; TRIPATHY, SARITA; KUMAR PATNAIK, PRASANT (2026). Figure 1.docx. figshare. Dataset. https://doi.org/10.6084/m9.figshare.31287844.v2 ( Acharya et al. 2026d) The project contains the following underlying data:
•
Figure 1.docx (Figure 1. Flow Chart of System Model) Data are available under the terms of the Creative Commons Attribution 4.0 International license (CC-BY 4.0).
6. Figshare: ACHARYA, MANAS RANJAN; TRIPATHY, SARITA; KUMAR PATNAIK, PRASANT (2026). Figure 2.docx. figshare. Dataset. https://doi.org/10.6084/m9.figshare.31287874.v1 ( Acharya et al. 2026e) The project contains the following underlying data:
•
Figure 2.docx (Figure 2. Basic diagram of F5GLO Model) Data are available under the terms of the Creative Commons Attribution 4.0 International license (CC-BY 4.0).
7. Figshare: ACHARYA, MANAS RANJAN; TRIPATHY, SARITA; KUMAR PATNAIK, PRASANT (2026). Figure 3.docx. figshare. Dataset. https://doi.org/10.6084/m9.figshare.31287880.v1 ( Acharya et al. 2026f) The project contains the following underlying data:
•
Figure 3.docx (Figure 3. Average and Maximum Latency Comparison) Data are available under the terms of the Creative Commons Attribution 4.0 International license (CC-BY 4.0).
8. Figshare: ACHARYA, MANAS RANJAN; TRIPATHY, SARITA; KUMAR PATNAIK, PRASANT (2026). Figure 4.docx. figshare. Dataset. https://doi.org/10.6084/m9.figshare.31288048.v1 ( Acharya et al. 2026g) The project contains the following underlying data:
•
Figure 4.docx (Figure 4. Peak Load handling: Maximum Latency Comparison) Data are available under the terms of the Creative Commons Attribution 4.0 International license (CC-BY 4.0).
9. Figshare: ACHARYA, MANAS RANJAN; TRIPATHY, SARITA; KUMAR PATNAIK, PRASANT (2026). Figure 5.docx. figshare. Dataset. https://doi.org/10.6084/m9.figshare.31288054.v1 ( Acharya et al. 2026h) The project contains the following underlying data:
•
Figure 5.docx (Figure 5. Latency Distribution: Comparison of Models) Data are available under the terms of the Creative Commons Attribution 4.0 International license (CC-BY 4.0).
10. Figshare: ACHARYA, MANAS RANJAN; TRIPATHY, SARITA; KUMAR PATNAIK, PRASANT (2026). Figure 6.docx. figshare. Dataset. https://doi.org/10.6084/m9.figshare.31288066.v1 ( Acharya et al. 2026i) The project contains the following underlying data:
•
Figure 6.docx (Figure 6. Throughput Analysis) Data are available under the terms of the Creative Commons Attribution 4.0 International license (CC-BY 4.0).
11. Figshare: ACHARYA, MANAS RANJAN; TRIPATHY, SARITA; KUMAR PATNAIK, PRASANT (2026). Figure 7.docx. figshare. Dataset. https://doi.org/10.6084/m9.figshare.31288099.v1 ( Acharya et al. 2026j) The project contains the following underlying data:
•
Figure 7.docx (Figure 7. Resource Utilization Comparison) Data are available under the terms of the Creative Commons Attribution 4.0 International license (CC-BY 4.0).
12. Figshare: ACHARYA, MANAS RANJAN; TRIPATHY, SARITA; KUMAR PATNAIK, PRASANT (2026). Figure 8.docx. figshare. Dataset. https://doi.org/10.6084/m9.figshare.31288135.v1 ( Acharya et al. 2026k) The project contains the following underlying data:
•
Figure 8.docx (Figure 8. PRA-Allocated Resources over Time) Data are available under the terms of the Creative Commons Attribution 4.0 International license (CC-BY 4.0).
13. Figshare: ACHARYA, MANAS RANJAN; TRIPATHY, SARITA; KUMAR PATNAIK, PRASANT (2026). Figure 9.docx. figshare. Dataset. https://doi.org/10.6084/m9.figshare.31288183.v1 ( Acharya et al. 2026l) The project contains the following underlying data:
•
Figure 9.docx (Figure 9. ANS-Network slice selection) Data are available under the terms of the Creative Commons Attribution 4.0 International license (CC-BY 4.0).
14. Figshare: ACHARYA, MANAS RANJAN; TRIPATHY, SARITA; KUMAR PATNAIK, PRASANT (2026). Algorithm 1.docx. figshare. Dataset. https://doi.org/10.6084/m9.figshare.31288345.v1 ( Acharya et al. 2026a) The project contains the following underlying data:
• Algorithm 1.docx (Algorithm 1: Predictive Resource Allocation (PRA)) Data are available under the terms of the Creative Commons Attribution 4.0 International license (CC-BY 4.0).
15. Figshare: ACHARYA, MANAS RANJAN; TRIPATHY, SARITA; KUMAR PATNAIK, PRASANT (2026). Algorithm 2.docx. figshare. Dataset. https://doi.org/10.6084/m9.figshare.31288360.v1 ( Acharya et al. 2026b) The project contains the following underlying data:
• Algorithm 2.docx (Algorithm 2: Latency-Aware Dynamic Routing (LADR) Data are available under the terms of the Creative Commons Attribution 4.0 International license (CC-BY 4.0).
16. Figshare: ACHARYA, MANAS RANJAN; TRIPATHY, SARITA; KUMAR PATNAIK, PRASANT (2026). Algorithm 3.docx. figshare. Dataset. https://doi.org/10.6084/m9.figshare.31288372.v1 ( Acharya et al. 2026c) The project contains the following underlying data:
• Algorithm 3.docx (Algorithm 3: Adaptive Network Slicing (ANS)) Data are available under the terms of the Creative Commons Attribution 4.0 International license (CC-BY 4.0).
