# Supplementary material for: Low-Latency Digital Health Framework for Rural Areas Leveraging Fog Computing and 5G
Source: F1000Res. 2026 May 4;15:661. [Version 1] doi: 10.12688/f1000research.178047.1 (PMC13554734; doi:10.12688/f1000research.178047.1)
Supplement: Peer review history

# Reviewer report: Reviewer response for version 1

**DOI:** 10.5256/f1000research.196385.r501193

**Published:** 2026-07-24

**Authors:** Jitendra Kumar Rout

**Affiliations:** 1 NIT Raipur, Raipur, India

**Summary**

 This manuscript proposes the F5GLO (Fog-5G Latency Optimization) framework to address the critical problem of communication latency in rural healthcare delivery. By integrating fog computing with 5G networks, the author demonstrates through simulation an 87% reduction in end-to-end latency compared to conventional cloud-based models. The three proposed algorithms—Predictive Resource Allocation (PRA), Latency-Aware Dynamic Routing (LADR), and Adaptive Network Slicing (ANS)—are clearly described and logically aligned with the latency minimization objective. The work is novel, well-motivated, and has direct practical implications for telemedicine and emergency services in underserved regions.

 Strengths

 -Clear problem definition with real-world relevance to rural healthcare disparities.

 -Sound theoretical foundation drawing on established fog computing and 5G literature.

 -Quantitative simulation results (latency distribution, throughput, energy efficiency) convincingly support the claimed improvements.

 -Well-structured algorithms with input/output specifications and stepwise logic.

**Minor Observations** (Not Requiring Changes)

 The following points are noted for the author’s awareness but do not affect the acceptance decision:

 -The mathematical formulation in Section 2.6 would benefit from an explicit equation for L_total; however, the conceptual description remains understandable.

 -Real-world validation in an actual rural testbed would strengthen future work, as the author correctly acknowledges in the conclusion.

 -A brief discussion of security and privacy considerations for patient data 

 Final Recommendation

 The paper is technically sound, well-written, and makes a meaningful contribution to digital health infrastructure research. It meets the standards for indexing. I recommend Acceptance without further revisions.

Is the work clearly and accurately presented and does it cite the current literature?

Yes

If applicable, is the statistical analysis and its interpretation appropriate?

Yes

Are all the source data underlying the results available to ensure full reproducibility?

Yes

Is the study design appropriate and is the work technically sound?

Yes

Are the conclusions drawn adequately supported by the results?

Yes

Are sufficient details of methods and analysis provided to allow replication by others?

Yes

Reviewer Expertise:

Applications of AI and ML, IoT, and NLP

I confirm that I have read this submission and believe that I have an appropriate level of expertise to confirm that it is of an acceptable scientific standard.

# Reviewer report: Reviewer response for version 1

**DOI:** 10.5256/f1000research.196385.r483176

**Published:** 2026-06-04

**Authors:** Chandra Kanta Samal

**Affiliations:** 1 University of Delhi, New Delhi, Delhi, India

Author’s research work on digital health system for rural areas using Fog Computing and 5G Technology; propose three algorithms: 1. Predictive Resource Allocation (PRA) method is presented in which dynamically allocates network resources based on predicted demand and current consumption patterns. 2. Latency-Aware Dynamic Routing (LADR) mechanism used to select the optimal routing path based on latency constraints. 3. Adaptive Network Slicing (ANS) mechanism, which dynamically assigns network slices based on system load and priority requirements. Author’s mention of the F5GLO model using Fog Computing and 5G technologies. The F5GLO simulation results in comparison with the Local Server Model and Traditional Cloud Model in the context of Average and Maximum Latency, Peak load handling, Maximum latency,

 Throughput, Efficiency, and Resource utilization. The F5GLO model proves its ability to significantly decrease the response time, with the maximum decrease of latency up to 87%. F5GLO is also characterized by better energy efficiency and high throughput in addition to relying on more powerful technology, such as fog computing and 5G technology, to improve the performance of the rural health system. Author’s is not mentioning the simulation configuration details for energy efficiency and not details discussing the real-world application. As per proposed algorithms and the suggested F5GLO model, simulation results to prove low latency due to

 improving the IoT-based service.

Is the work clearly and accurately presented and does it cite the current literature?

Yes

If applicable, is the statistical analysis and its interpretation appropriate?

Yes

Are all the source data underlying the results available to ensure full reproducibility?

Yes

Is the study design appropriate and is the work technically sound?

Yes

Are the conclusions drawn adequately supported by the results?

Yes

Are sufficient details of methods and analysis provided to allow replication by others?

Yes

Reviewer Expertise:

Wireless Networks, Brain Networks, Social Networks etc

I confirm that I have read this submission and believe that I have an appropriate level of expertise to confirm that it is of an acceptable scientific standard.
